# Gaze Information Channel in Cognitive Comprehension of Poster Reading

**DOI:** 10.3390/e21050444

**Published:** 2019-04-28

**Authors:** Qiaohong Hao, Mateu Sbert, Lijing Ma

**Affiliations:** 1 College of Intelligence and Computing, Tianjin University, Yaguan Road 135, Tianjin 300350, China; 2 Institute of Informatics and Applications, University of Girona, 17003 Girona, Spain

**Keywords:** information channel, eye tracking, cognitive comprehension, entropy, mutual information

## Abstract

Today, eye trackers are extensively used in studying human cognition. However, it is hard to analyze and interpret eye movement data from the cognitive comprehension perspective of poster reading. To find quantitative links between eye movements and cognitive comprehension, we tracked observers’ eye movement for reading scientific poster publications. We model in this paper eye tracking fixation sequences between content-dependent Areas of Interests (AOIs) as a Markov chain. Furthermore, we use the fact that a Markov chain is a special case of information or communication channel. Then, the gaze transition can be modeled as a discrete information channel, the gaze information channel. Next, some traditional eye tracking metrics, together with the gaze entropy and mutual information of the gaze information channel are calculated to quantify cognitive comprehension for every participant. The analysis of the results demonstrate that the gaze entropy and mutual information from individual gaze information channel are related to participants’ individual differences. This is the first study that eye tracking technology has been used to assess the cognitive comprehension of poster reading. The present work provides insights into human cognitive comprehension by using the novel gaze information channel methodology.

## 1. Introduction

As we all know, the eye is an important organ of the human being. It is often said that the eyes are the window of the soul, reflecting the thoughts of us human beings, and revealing the way in which the participants observe the scene. With more and more researchers using eye tracking technology as a research tool, eye tracking is a promising method in academic and industrial research. It has the potential to provide insights into a lots of issues in the visual and cognitive fields: education [1,2,3], medicine [4,5,6,7], assistive technology for people with a variety of debilitating conditions [8,9,10], better interface design [11,12,13], marketing and media [14,15,16]. Furthermore, as an important psychological experiment research method, eye movement provides a new perspective and way for educational technology research [17,18,19]. Actually, eye tracking has always been an important human–computer interaction method for making decisions [20,21,22].

Importantly, research based on the idea of using eye tracking as an instructional tool is still in its infancy. There is an urgent need to quantitatively compare eye movement metrics [23]. Several eye tracking metrics have been developed. The first are scanpaths, represented by an ordered sequence of fixations, for which vector and string-based editing methods have been developed to compute similarity [24,25,26,27]. The second are heatmaps, represented by Gaussian Mixture Models (GMMs) indicating the frequency (or probability) of fixation location [28,29]. A third type of sequential fixation pattern analysis is the transition matrix, which is rarely used as a quantitative measure [30]—see also the recent survey by Shiferaw et al. [31]. In this paper, we model eye tracking fixation sequences of Areas of Interests (AOIs) as a Markov chain. Furthermore, we model the gaze transition as a gaze information channel, introduced in [32]. We extend here the gaze information channel in [32] with a more complete description and interpretation of the metrics of the channel, and by showing how it is well adapted for clustering, allowing thus to analyze collective behavior. We also notice the importance of normalization of mutual information that was not considered in [32], when comparing different channels’ data.

While efforts are made to teach the elements of writing a scientific article in many graduate school curricula, much less attention is paid to teaching those skills necessary to read scientific posters, even though these arguably are the most common and most rapid ways to disseminate new findings. Especially for graduate students who are committed to scientific research, reading related research papers is an extremely important skill, and it is also a reflection of research ability. Actually, posters provide a coherent and efficient way to convey core ideas expressed in scientific papers, as described in [33]. Thus, how to quickly grasp the core idea of a scientific paper is also an essential ability for them. Furthermore, poster as a form of academic expression represents a concise and visual summary of one’s research. Its purpose is to be accessible and to drive attention to the research, and get the main point of the research across to as many people as possible through a concise and artistically attractive manner [34,35,36]. In other words, poster is one style of the most vivid and short scientific papers, which can best reflect a reader’s scientific reading skills and thinking process. Qiang et al. [33] used probabilistic graphical models to learn scientific poster design patterns, from existing posters, and they proposed an algorithm that considered both information conveyed and aesthetics to generate the poster layout. They used subjective evaluation of readability, informativeness and aesthetics to compare different designs of a poster. However, there is to our knowledge no study that has investigated by using eye tracking the cognitive comprehension of poster reading. Is it possible to use eye tracking data to quantify cognitive comprehension during reading poster from participants? This article reports on our efforts to answer this question.

**Figure 1:**
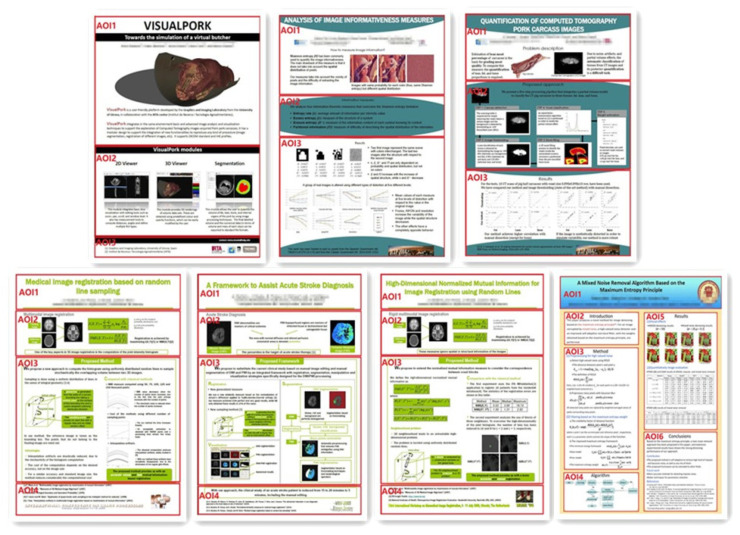
The poster materials used in our eye tracking experiment. From left to right, in top row, posters 1, 2, and 3, in bottom row, posters 4, 5, 6, and 7. Marked in red, the Areas of Interests (AOIs) in which each poster is divided, and that are not displayed to the participants.

Ten participants’ eye movement data of reading published posters were recorded individually using an eye tracker under controlled laboratory conditions. The tested posters are divided into content-dependent Area of Interests (AOIs), following the sections of a poster defined by the authors, as shown in Figure 1. The gaze information channel was used to analyze and interpret the eye tracking data. Some traditional eye tracking metrics, together with the gaze entropy and mutual information, are calculated to quantify cognitive comprehension of poster reading for every participant.

## 2. Background

Ponsoda et al. [37] introduced probability vectors and transition matrices by classifying the directions of saccade. Interestingly, their matrices were based on transition between the eight main saccade directions rather than between the Areas of Interests (AOIs), which are now more commonly used. Although Ponsoda et al. compared the matrices with a statistical method, they did not model the sequence of saccade directions as a Markov chain.

Ellis and Stark [38] compared the airline pilot transition matrices by dividing cockpit display traffic information (CDTI) into eight AOIs. They introduced first-order (fixation) transition matrices and converted them to conditional probability matrices. Then, conditional entropy was calculated using the conditional probability, or transition, matrices. Its value provided a measure of the statistical dependency in the spatial pattern of fixations represented by the transition matrix.

Liechty et al. [39] used Hidden Markov Models (HMMs) to distinguish between local and global visual attention states in eye movement data. Instead of applying the transition matrix Markov model as we do in this paper, they used HMMs to distinguish between fixations, similar to Velichkovsky et al. [40], who proposed classifying attention as ambient or focal.

Hwang et al. [41] did not construct a transition matrix between AOIs or in a grid, but considered position translation within a generated saliency map for a given scene, and introduced transitional semantic guidance calculations to evaluate gaze transition. This method can be seen as a hybrid between the transformation matrix construction and the scan path comparison where the transition matrices were replaced by semantic maps. Because the saliency maps generated by each scene may be different, one drawback of this approach is the comparison between scenes. This problem can be solved by building content-independent transition matrices.

Bonev et al. [42] built a Markov chain between the nodes of a regular grid matrix, the elements of the matrix being the normalized mutual information defined by the covariances of the Gaussian distribution of the attributes of the image. They obtained the equilibrium distribution of this Markov chain and defined the entropy of this equilibrium distribution as the complexity of the image. Thus, this complexity measure was defined independently of any observational task, only depending on grid and Gaussian distribution of attributes of the image. Then, Bonev et al. studied the correlation of this complexity with the sequences of long and short saccades. In our case, Markov chain transition probabilities matrix is defined from the observation trajectories, and thus it depends on the task.

Besag and Mondal [43] verified the feasibility of modeling gaze transition as a first-order Markov process. According to modeling eye movement transitions between areas of interest (AOIs) as a Markov chain, Krejtz et al. [44,45] calculated stationary entropy $H_{s}$ and transition entropy $H_{t}$ to measure the complexity of the Markov process. Raptis et al. [46] divided the images into three AOIs and used the gaze transition entropy proposed by Krejtz et al. [44] as a tool to quantify differences on visual search patterns among individuals within visual pattern recognition tasks of varying complexity.

## 3. Information Measures and Information Channel

In this section, we briefly introduce the most basic information measures of information theory and main elements of the information channel [47]. Since its inception by Shannon [48], information theoretic measures and concepts, which include as one of the main tools the information or communication channel, have been successfully used in many fields. For their application in visualization, image processing, and pattern recognition, see [49,50].

### 3.1. Basic Information-Theoretic Measures

Let *X* be a discrete random variable with alphabet **X** and probability distribution {p(x)}, where p(x)=Pr{X=x},x∈X. In this paper, {p(x)} will be denoted by p(X).

The *entropy* H(X) of a discrete random variable *X* is defined by

(1) $$H\left(X\right)=-\sum_{x\in X}p\left(x\right)\log p\left(x\right),$$

where the summation is over the corresponding alphabet and the convention 0log0=0 is taken. In this paper, logarithms are taken in base 2 and, as a consequence, entropy is expressed in bits. The entropy H(X) gives the average uncertainty (or amount of information) of a random variable *X*.

The *joint entropy* H(X,Y) of a pair of discrete random variables *X* and *Y* with a joint probability distribution p(X,Y)={p(x,y)} is defined by

(2) $$H\left(X,Y\right)=-\sum_{x\in X}\sum_{y\in Y}p\left(x,y\right)\log p\left(x,y\right),$$

where p(x,y)=Pr[X=x,Y=y] is the joint probability of *x* and *y*.

The *conditional entropy* H(Y|X) of a random variable *Y* given a random variable *X* is defined by

(3) $$\begin{array}{cc} H(Y|X) & =\sum_{x\in X}p\left(x\right)H\left(Y|X=x\right)=\sum_{x\in X}p\left(x\right)\left(-\sum_{y\in Y}p\left(y|x\right)\log p\left(y|x\right)\right)\\ & =-\sum_{x\in X}\sum_{y\in Y}p\left(x,y\right)\log p\left(y|x\right), \end{array}$$

where p(y|x)=Pr[Y=y|X=x] is the conditional probability of *y* given *x*. H(Y|X) measures the average uncertainty associated with *Y* if we know the outcome of *X*.

The *mutual information* I(X;Y) between two random variables *X* and *Y* is defined by

(4) $$\begin{array}{cc} I(X;Y) & =H(X)+H(Y)-H(X,Y)\\ & =H(X)-H(X|Y)=H(Y)-H(Y|X)\\ & =\sum_{x\in X}\sum_{y\in Y}p\left(x,y\right)\log\frac{p(x,y)}{p(x)p(y)}. \end{array}$$

Mutual information (MI) represents that knowledge of *Y* decreases the uncertainty of *X*, and vice versa. I(X;Y) is a measure of the shared information or dependence between *X* and *Y*.

**Figure 2:**
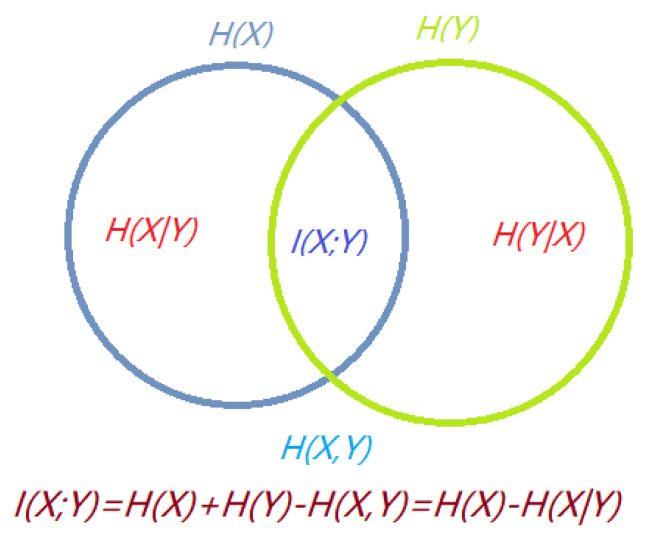
The information diagram represents the relationship between Shannon’s information measures.

The relationship between Shannon’s information measures can be illustrated by a Venn diagram, as shown in Figure 2. The information diagram represents the relationship between Shannon’s information measures. Observe that I(X;Y) and H(X,Y) are represented, respectively, by the intersection and the union of the information in *X* (represented by H(X)) with the information in *Y* (represented by H(Y)). H(X|Y) is represented by the difference between the information in *X* and the information in *Y*, and vice versa for H(Y|X).

### 3.2. Information Channel

**Figure 3:**
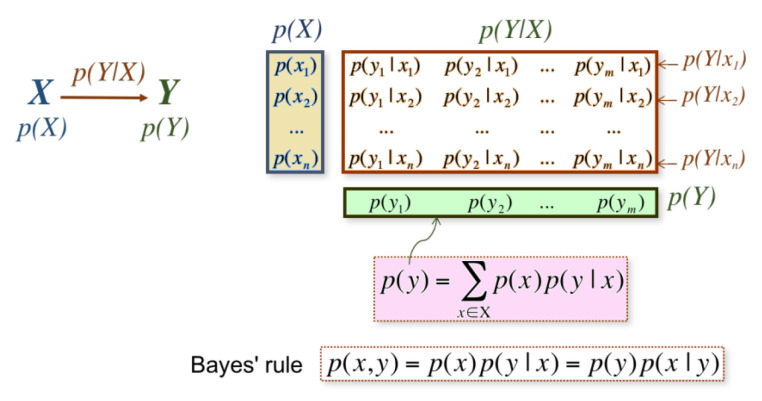
The main elements of an information channel.

*Communication or information channel* is a system in which the output depends probabilistically on its input [47,51]. The conditional entropy and mutual information can be thought of in terms of a communication or information channel X→Y whose output *Y* depends probabilistically on its input *X*. This information channel is characterized by a transition probability matrix which determines the conditional distribution of the output given the input [47,51]. Given that *X* and *Y* are two random variables, we can establish an information channel between *X* and *Y*. The diagram in Figure 3 shows the main elements of the information channel:

- p(X) and p(Y) represent the probability distributions of input and output variables *X* and *Y*, respectively.
- Probability transition matrix p(Y|X) composed of conditional probabilities p(y|x), which gives the output distribution p(Y) given the input distribution p(X). Each row of p(Y|X) can be seen as a probability distribution, denoted by p(Y|x).

All of these elements are connected by the Bayes theorem relating marginal probabilities p(X) and p(Y), conditional probabilities p(y|x) and p(x|y), and joint probabilities p(x,y): p(x,y)=p(x)p(y|x)=p(y)p(x|y).

## 4. Method

In this section, we introduce how to set up the gaze information channel.

### 4.1. Gaze Information Channel

Gaze information channel has been proposed in our previous work [32]. A Markov chain is a stochastic model that describes a series of possible events $X_{1},X_{2},X_{3},\ldots$, in which the probability of each event depends only on the state of the previous event, or Markov property. If the state space is finite, the transition probability distribution can be represented by a matrix, called the transition matrix. A time-invariant Markov chain is characterized by its initial state and a probability transition matrix $P=[p_{ij}]$ [52]. A Markov chain $\left\{X_{i}\right\}$ is fully determined by the initial state $X_{0}$ and the transition matrix $P=[p_{ij}]$, $p_{ij}=Pr\left\{X_{n+1}=j|X_{n}=i\right\}$, i,j∈{1,2,…,s}, where *s* is the number of states.

A Markov chain is said to be irreducible if its state space is a single communicating class; in other words, if it is possible to get to any state from any other state. It is aperiodic if all its states are aperiodic, that is, the return to any state is not constrained to a number of steps multiple of any integer >1. An irreducible and aperiodic Markov chain has a positive stationary distribution, the stationary distribution is unique, and from any starting distribution, the distribution of $X_{n}$ tends to the stationary distribution as n→∞. The stationary distribution can be calculated by Equation (5):

(5) πP=π.

The stationary distribution represents the frequency of visits of each state.

In this paper, we divide a tested poster into *s* content-dependent AOIs. The set of AOIs can be represented as S={1,2,…,s}, and the gaze switching process can be described as a stochastic process $\{X_{t}\}\;,t=1,2,\ldots ,n$, $x_{1},x_{2},\ldots ,x_{n}\in S$. In [44], the Markov property has been tested. Once the stochastic process is modeled as a Markov process, we obtain the transition matrix $P={\left[p_{ij}\right]}_{s\times s}$ and the stationary or equilibrium probability π.

Similar to the work in [53] in the 3D scene visibility context, and as proposed in [32] to study Van Gogh’s painting, we extend the Markov chain model [44] for gaze transitions when reading posters to an information channel, X→Y, where *X* and *Y* are discrete random variables with alphabet X=Y=1,2,…,s, corresponding to the AOIs. In this case, input variables p(X) and output variables p(Y) of gaze information channel represent the same regions with the same probabilities π. The conditional probability p(j|i) in the gaze information channel corresponds to the $p_{ij}$ of transition matrix *P* in the Markov chain. Contrary to the case in [32], where the AOIs where arbitrarily fixed for a painting, we consider the AOIs in the posters as being defined by the authors in their poster design, that is, the different sections that are contained in a poster.

The basic elements of the *gaze information channel* are thus the following ones:

- The conditional probability p(j|i) is given by $p_{ij}$, which represents the estimated probability of transitioning from $i_{th}$ AOI to any $j_{th}$ AOI given $i_{th}$ AOI as the starting point. Matrix elements $p_{ij}$ are set to the number of transitions from $i_{th}$ source AOI to $j_{th}$ destination AOI for each participant and then the matrix is normalized relative to each source AOI (i.e., per row), $p_{ij}=\frac{n_{ij}}{\sum_{j=1}n_{ij}},i,j\in S$. Conditional probabilities fulfill $\sum_{j\in Y}\;p\left(j|i\right)=1$,∀i∈X, that is, $\sum_{j=1}^{s}p_{ij}=1,\forall i\in \left\{1,\ldots ,s\right\}$.
- The marginal probabilities of input *X* and output *Y*, p(i) and p(j), are both given by the stationary probability π, $\pi =(\pi_{1},\pi_{2},\ldots ,\pi_{s})$, giving the frequency of visits of each AOI.

### 4.2. Entropy and Mutual Information in Gaze Information Channel

From the previous definitions and Equations (1)–(4), Shannon’s information measures can be defined for the gaze information channel. We first introduce the entropy of the input (and also output), random variables with stationary distribution,

(6) $$H_{s}=H\left(X\right)=H\left(Y\right)=-\sum_{i=1}^{s}\pi_{i}\log\pi_{i}.$$

As the equilibrium distribution represents the average number of visits in each AOI, $H_{s}$ indicates the average uncertainty of gaze position between the AOIs. Low $H_{s}$ values of stationary entropy means that the observer prefers some AOIs over the other ones, while high values mean that visual attention is balanced between AOIs.

The conditional entropy of ith row, H(Y|i), is defined as

(7) $$H\left(Y|i\right)=-\sum_{j=1}^{s}p_{ij}\log p_{ij}.$$

It gives the uncertainty that the next fixation would be the $j_{th}$ AOI if it were presently in the $i_{th}$ AOI.

The conditional entropy $H_{t}$ of the information channel is the average of row entropies

(8) $$H_{t}=H\left(Y|X\right)=\sum_{i=1}^{s}\pi_{i}H\left(Y|i\right)=-\sum_{i=1}^{s}\pi_{i}\sum_{j=1}^{s}p_{ij}\log p_{ij}.$$

It represents the average uncertainty of a transition between two areas of interest, or average uncertainty that remains about the destination AOI when the source AOI is known.

The joint entropy H(X,Y) of the information channel is the entropy of the joint distribution of *X* and *Y*

(9) $$\begin{array}{c} H\left(X,Y\right)=H\left(X\right)+H\left(Y|X\right)=H_{s}+H_{t}=\sum_{i=1}^{s}\sum_{j=1}^{s}\pi_{i}p_{ij}\log\pi_{i}p_{ij} \end{array}$$

and gives the total uncertainty of the channel. The mutual information of $i_{th}$ row, I(i;Y), is given by

(10) $$I\left(i;Y\right)=\sum_{j=1}^{s}p_{ij}\log\frac{p_{ij}}{\pi_{j}}$$

and represents the degree of correlation between AOI *i* and all the AOIs. The measures I(i;Y) and H(Y|i) show in general opposite behavior patterns. A high value of H(Y|i) represents a high degree of uncertainty about next area of interest, while a high value of I(i;Y) indicates the next AOI is known with high probability.

The mutual information I(X;Y) is given by

(11) $$\begin{array}{c} I\left(X;Y\right)=H\left(X\right)+H\left(Y\right)-H\left(X,Y\right)=\sum_{i=1}^{s}\pi_{i}I\left(i\;;Y\right)\\ =\sum_{i=1}^{s}\sum_{j=1}^{s}\pi_{i}p_{ij}\log\frac{p_{ij}}{\pi_{j}} \end{array}$$

and represents the total correlation, or information shared, between the AOI’s.

## 5. Experiment and Data Collection

### 5.1. Materials

To set up the test, we selected, with permission of authors, seven image processing research posters as the testing materials. All students participating in the experiment had sufficient knowledge background to understand the tested posters, and confirmed that they had never seen the materials before. The posters AOIs followed the sections defined by the authors of the posters. For the sake of display in this paper, we combine all tested posters into one image, Figure 1. In order to make the reader of this paper more aware of the AOIs, we use the red block diagram to mark them in Figure 1, but in the eye tracking experiment, the red block diagram will not be displayed to participants. Moreover, we blurred the author and institutional information.

### 5.2. Participants

A total of 10 master in computer science students (male: 5, female: 5) from Tianjin University (Tianjin, China) participated in the eye tracking experiment. Their ages range from 22 to 28 years (average: 23.75, standard deviation: 1.5). All the participants can understand well English and have normal color vision. They had enough background to understand the posters, although they had not seen them before. Before the experiment, all participants signed a consent form.

### 5.3. Procedure

Equipment calibration was completed prior to the experiment. Then, the participants were instructed to view the poster as though they were reading papers as usual. These tested materials were presented for 60 s. Everyone was seated in an office chair, and asked to lean forward to rest his/her chin comfortably, with his/her head is 60 cm distant from the computer screen. During the eye tracking, there was no interaction between the operator and the participants. The posters were presented one after another. After the experiment, each participant was asked to review individually the poster once again and explain the core idea of the tested poster.

### 5.4. Apparatus and Site Setting

The SMI iViewETG2.7 eye tracker (Sensomotoric Instruments, Teltow, Germany) and BeGaze 3.7 software (Sensomotoric Instruments, Teltow, Germany) were utilised for data collection and for computing eye gaze metrics. The participants wore the eye tracker and looked at the high resolution (1920×1080) 24 inch LCD monitor that displayed the tested posters. The experiment was conducted in a quiet room. The curtains of the room were pulled to avoid uncontrollable light and reflection.

### 5.5. Data Collection

Eye movements were recorded with an SMI iViewETG2.7 eye tracking system. The raw video data was produced by the iViewETG2.7, and then video data was input to the eye tracking analysis software BeGaze 3.7 to edit AOIs, produce some visualizations (scanpath, heatmap and the bin charts of AOIs), and generate a series of fixations. Each fixation contains four parameters: the start time, the duration, and the X and Y position on the screen. The following analysis is based on this format of eye tracking data.

## 6. Results Analysis

### 6.1. Traditional Metrics

**Figure 4:**
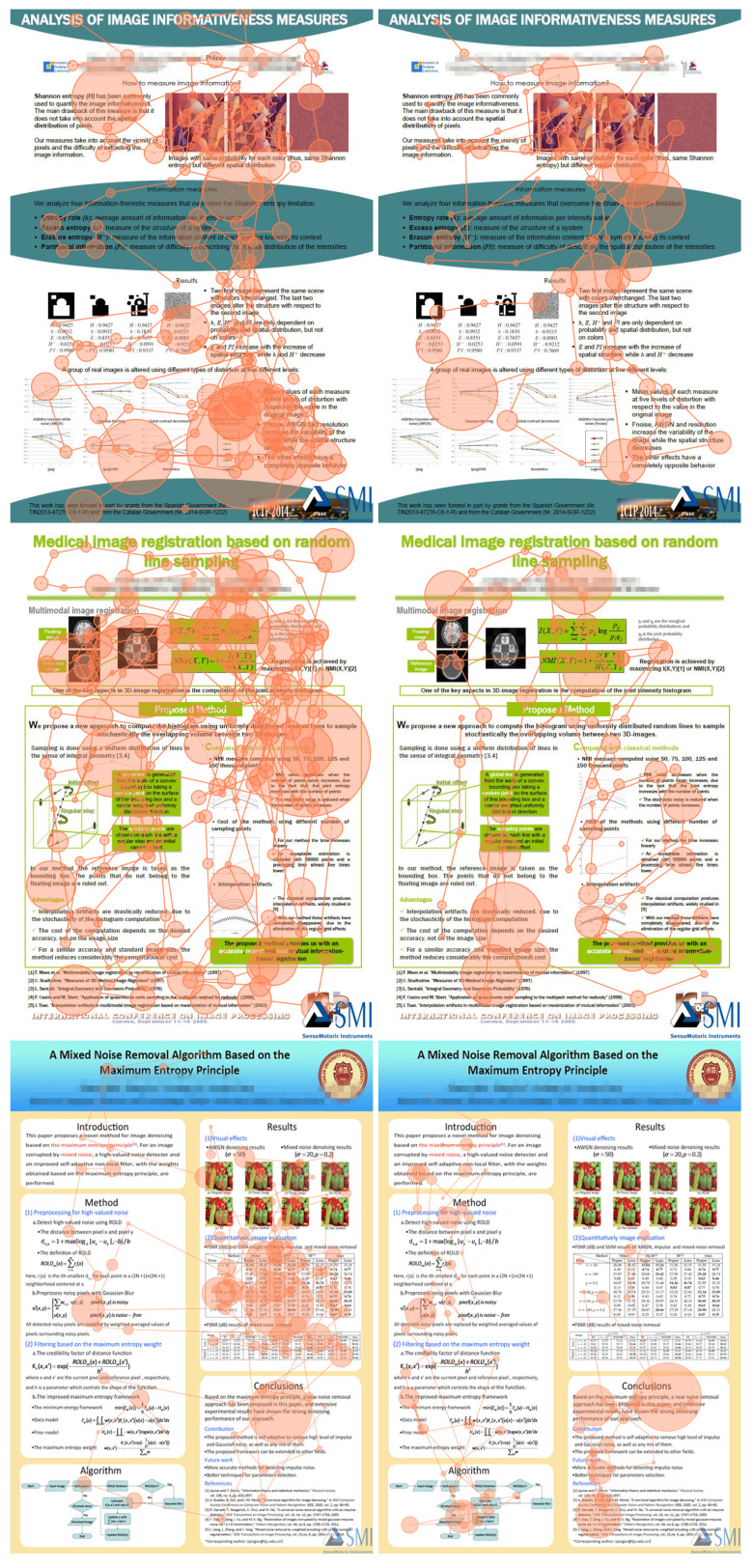
The scanpaths for participant 2 (**left**) and participant 5 (**right**) for posters 2, 4 and 7.

We first applied some traditional metrics and visualizations on the collected eye tracking data. These include: scanpaths with fixation count, heatmaps, bin charts, and the bin charts of AOIs. Since the limited space of this article, we just take participants 2 and 5, selected at random, as an example to present the result analysis for the convenience of display. Figure 4 shows the scanpaths of the tested posters from participant 2 and participant 5, respectively. The diameter of fixations, for all scanpaths, is set as 80 px = 500 ms. For the sake of space, in this section, we focus on only two participants and three posters based on the different number of AOIs to show the analysis of the results. Figure 4 left shows the scanpaths of participant 2; we can observe that the participant 2 is more focused on the result section of these posters because there are more fixations and duration in the image and table section. In contrast, the scanpaths of Figure 4 right of the participant 5 is free and more random. We can observe that this participant is not interested in the image and table area of the results section. Participant 5 is more focused on the overall reading and understanding.

**Figure 5:**
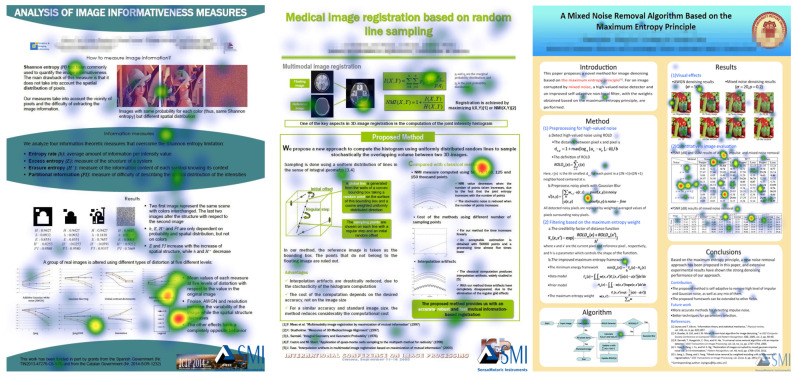
The heatmaps for participant 2 for posters 2, 4 and 7.

**Figure 6:**
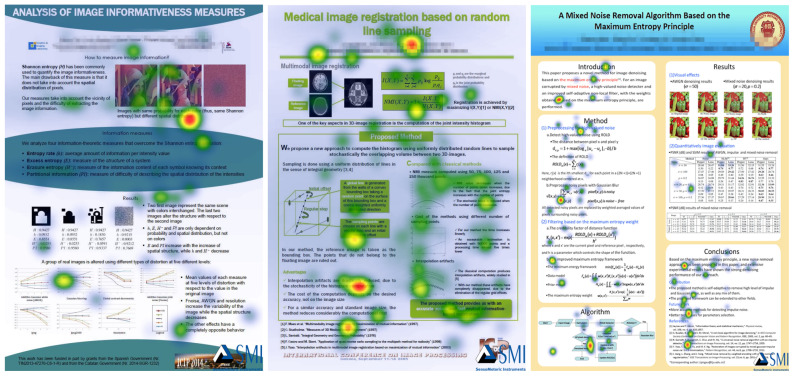
The heatmaps for participant 5 for posters 2, 4 and 7.

Figure 5 and Figure 6 present the heatmaps, from participant 2 and participant 5, respectively. Obviously, the result of the heatmap is similar to the result of the scanpath, as it is another representation of the same data. We can compare in Figure 5 and Figure 6 the participants’ attention distribution within the poster. Observe that ranges are not unified, thus the fixation times should be compared in Figure 4. The heatmap of the participant 2 shows that the participant 2 is more focused on the result section of the posters. In contrast, the heat map of Figure 6 of the participant 5 is free and more discrete, and focused on the text. We can see that participant 5 is not interested in the image and table detail area of the posters.

**Figure 7:**
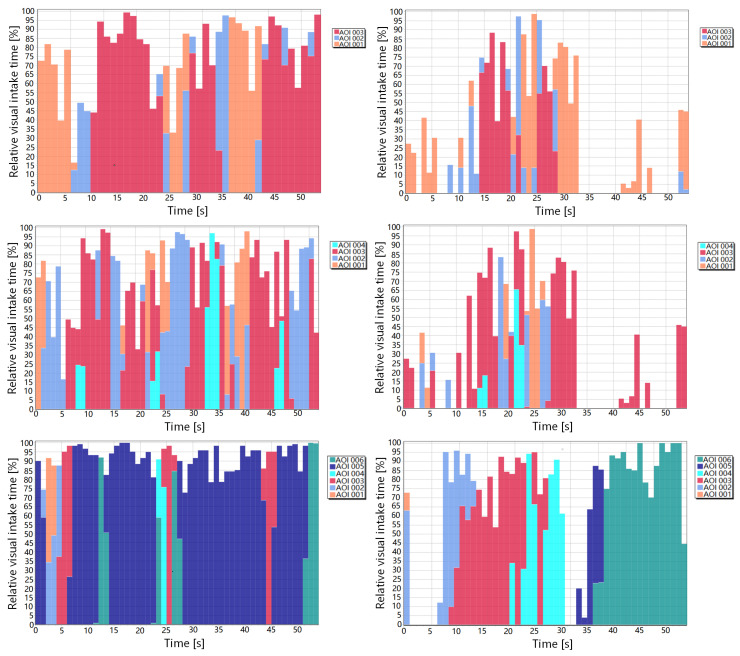
The AOI bin charts for participant 2 (**left**) and participant 5 (**right**) for posters 2, 4 and 7.

Figure 7 presents the bin charts of AOIs from participant 2 and participant 5, respectively. It shows the relative visual intake time of which AOI the observer falls on at each time. We can find that participant 2 prefers AOI 3 (results section) for posters 2 and 4, and AOI 5 (results section) for poster 7. The left bin charts show a large number of red areas for posters 2 and 4 (AOI 3) and blue areas for poster 7 (AOI 5), which are the AOIs corresponding to the result part in the test posters. Participant 5 is more focused too on AOI 3 for posters 2 and 4 (results section), as the red area from AOI bin chart is large in Figure 7 right, but it is focused on AOI 3 (method section) and AOI 6 (conclusions section) for poster 7 because the red area and cyan area from AOI bin chart is very large. This shows there can be individual differences between participants in reading the same poster.

### 6.2. Entropy and Mutual Information in Gaze Information Channel

We consider each poster divided into content-dependent AOIs. As described in Section 4, we compute the entropy and mutual information of the gaze information channel to quantify cognitive comprehension for each participant.

#### 6.2.1. Transition Matrices

In order to better understand the process of participant’s eye movement, we first analyze the gaze transition matrix when the participant views the tested posters. Table 1 provides the transition matrix before normalization of three tested posters (posters 1, 2, 3, with 3 AOIs) for all participants. That is, we accumulate in a single matrix all the transitions by all participants for these three posters. It can be observed that there are about 1200 fixations in total in the transition matrix, and the numbers (bold gray value in table) on the diagonal of the transition matrix are larger. This is consistent with a common sense interpretation, as the participant, before shifting to another area, will explore the area it is in until he/she has an understanding of it. Thus, participants’ cognitive process creates these transition matrix data shown in Table 1. Similarly, Table 2 shows the transition matrix before normalization of three tested poster (posters 4,5,6, with four AOIs) by all participants.

#### 6.2.2. Comparison between Two Participants for Poster 7

**Figure 8:**
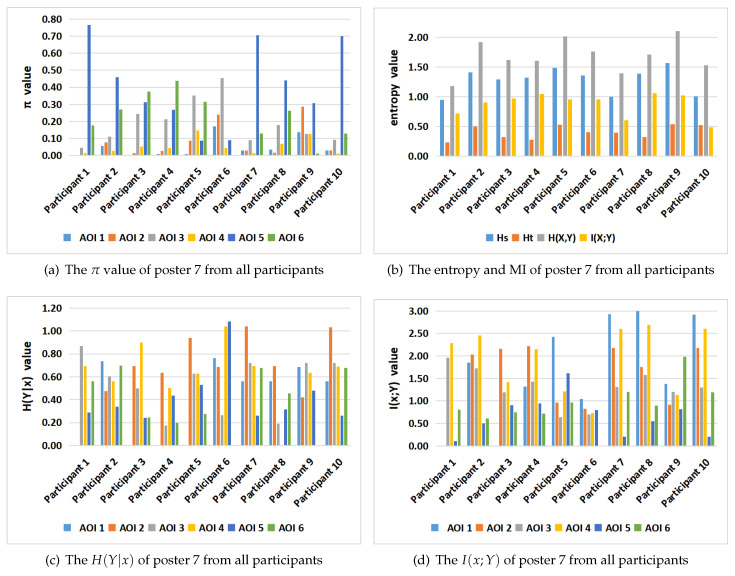
The channel measures for all participants and poster 7. From left to right and top to down, the equilibrium distribution *π* (**a**), *H_s_*, *H_t_*, *H*(*X*,*Y*), *I*(*X*;*Y*) (**b**), H(Y|x) (**c**), and *I*(*x*;*Y*) (**d**). AOI1 is the title section, AOI2 is the intro section, AOI3 is the method section, AOI4 is the algorithm section, AOI5 is the results section, and AOI6 is the conclusions section.

Here, we show first in Figure 8 the results for poster 7 for all participants and then we compare more finely for participants 2 and 5. Observe from Figure 8a that the AOIs more visited by all participants are AOI5 (results section), AOI6 (conclusions section), and AOI3 (method section), although the most visited area depends on the participant. The majority of participants prefer, or visit it often, AOI5 (results section), others AOI6 (conclusions section), and finally others AOI3 (method section). Figure 8b shows the main measures of the channel for each participant, some of them are similar for several participants, although from Figure 8c,d, we can conclude that the exploration strategy can be in general different for each participant.

**Figure 9:**
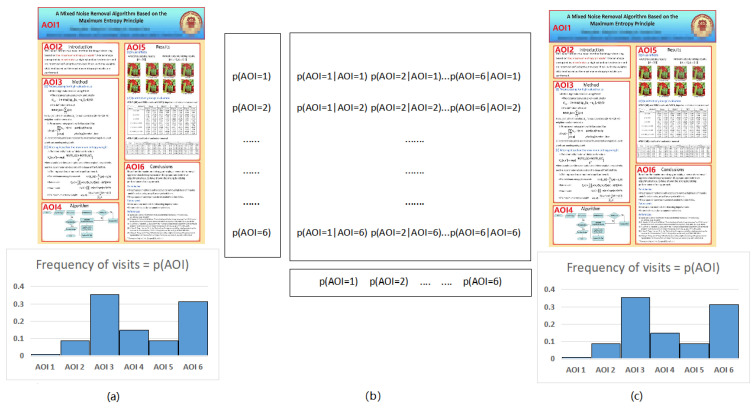
The gaze channel for participant 5 and poster 7, between the AOIs with equilibrium distribution p(AOI) (**a**) and (**c**), and with conditional probabilities (**b**). The probability distributions values are found in Table 3 and Table 4.

Next, Table 3 shows the transition probabilities of the participants 2 and 5 for the poster with more areas of interest, poster 7 with six AOIs. See Figure 9 for an illustration of the gaze channel for participant 5. Observe that, in Table 3, the values of $p_{ii}$ are the highest transition probabilities, which is consistent with the above transition matrix analysis. This is similar to *The tempest* painting example presented in [44]. As observed before, this means that, before switching to another AOI, the observer firstly moves the gaze within the current AOI. As shown in Table 3, we can clearly find that there is no direct transition between AOI 2 and AOI 6 when viewing the tested poster. The reason might be that the AOI 2 (introduction section of the poster) is far apart from AOI 6 (the conclusion section of the poster).

**Figure 10:**
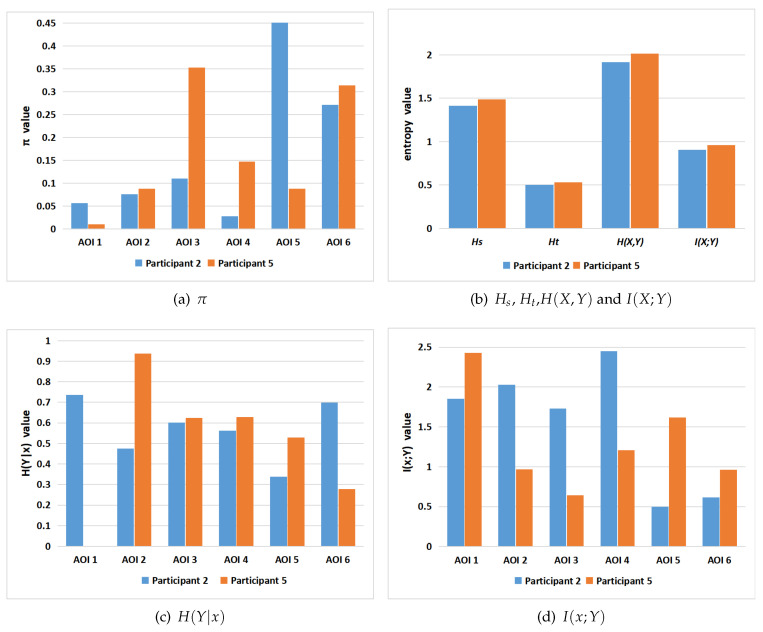
From left to right and top to down, the equilibrium distribution *π* (**a**), *H_s_*, *H_t_*, *H*(*X*,*Y*), *I*(*X*;*Y*) (**b**), H(Y|x) (**c**), and *I*(*x*;*Y*) (**d**) in gaze information channel of participants 2 and 5 for poster 7. The numerical values are found in Table 4.

Table 4 and Figure 10 show the values for the equilibrium distribution, $H_{s}$, $H_{t}$, H(Y|x), H(X,Y), I(X;Y) and I(x;Y), for the gaze information channel for participants 2 and 5. The gaze entropy $H_{s}$ is the entropy of the equilibrium distribution π, which indicates how frequently each AOI is visited. Note that currently in our gaze channel model, as in Markov chain model, we do not support fixation time, thus number of visits does not directly translate into spent time, although it can be considered as an approximation. From Table 4 and Figure 10, we can find that the AOIs that the participants prefer, AOI 5 (results section) for participant 2, and AOI 3 (method section) and AOI 6 (conclusions section) for participant 5, have the larger equilibrium distribution $\pi_{i}$ value. This is consistent with Figure 7 charts for poster 7. A higher value of $H_{s}$ means that the participant visited more equally each AOI. A lower value of $H_{s}$ is obtained when the number of visits in each AOI is not balanced, possibly because the participant spent more time concentrated on a certain region. It can be seen from Table 4 and Figure 10 that the entropy $H_{s}$ of the participant 5 is greater than for the participant 2. This means that the participant 5 pays more attention to overall reading and spent time more equally among AOIs than the participant 2. This conclusion is consistent with the previous scanpath analysis from Figure 4.

$H_{t}$ reflects the randomness of gaze transition among the different AOIs. Higher $H_{t}$ values mean that there are frequent transition among AOIs, while lower $H_{t}$ values indicate more careful observation of AOIs [44]. H(Y|i) measures the randomness of the gaze transfer from the *i*-th AOI. A lower value of H(Y|i) indicates that the observer is more clear about the next AOI in the following view. It may also represent that the *i*-th AOI provides the observer with significant clues to understand the test poster. From Table 4 and Figure 10, we can find that, for participant 2, H(Y|1) has the highest value, which means that when in AOI1 (title section of the poster), the observer moves randomly (or evenly) towards any of the other neighbour AOIs. For participant 5, H(Y|2) has the highest value, which indicates that this participant moves evenly from AOI 2 (intro section) to any AOI of the poster. Moreover, we can also see that I(3;Y) has the lowest value, which represents that the information shared between AOI3 (method section) and all the AOIs is minimum. $H\left(X,Y\right)=H_{s}+H_{t}$ measures the total uncertainty, or total randomness of fixations distribution and gaze transition. The lowest value of H(X,Y) is obtained when the participant 2 views the poster. Compared with the participant 5’s scanpath in Figure 4, the scanpath with lowest H(X,Y) has higher fixation length and less gaze transitions.

As expected, we can observe in Table 4 and Figure 10 that I(i;Y) and H(Y|i) show in general opposite behavior. Higher values of I(i;Y) correspond to lower values of H(Y|i) and viceversa. The values of I(4;Y) for participant 2 and I(1;Y) for participant 5 are higher than the other values of I(i;Y). This indicates that next AOIs when leaving AOI4 (algorithm section) for the participant 2, and leaving AOI1 (title section) for participant 5, were well defined, as a high value of I(i;Y) means that the next AOI is known with high probability. This behavior can be re-confirmed in the corresponding scanpaths in Figure 4. Furthermore, from Table 4 and Figure 10 we can see that participant 5 has the highest I(X;Y) value. Mutual information I(X;Y) expresses the degree of dependence between the AOIs. It might mean that participant 5 has a more precise strategy or more clues in exploring the tested poster. However, this is in apparent contradiction to the fact that total uncertainty of participant 5 is higher than for participant 2. To be able to compare the mutual information between the two participants, we should first normalize it. Several normalization proposals exist in the literature [54]. If we consider for instance the one defined in [47] as a correlation coefficient $\rho =\frac{I(X;Y)}{H(X)}=\frac{I(X;Y)}{H_{s}}$, the value of ρ for participant 2 is 0.643, and for participant 5 is 0.644, practically the same. Thus, in this case, we can not discover any difference based on mutual information.

#### 6.2.3. Averaging Results for All Posters and Participants

We can find in the Appendix the Table A1, Table A2, Table A3, Table A4, Table A5 and Table A6, with the values for all participants and posters of I(X;Y), $H_{s}$, $H_{t}$ and H(X,Y), and I(X;Y) normalized by $H_{s}$ and H(X,Y), respectively. For instance, Table A1 lists the mutual information I(X;Y) of all participants when they view all tested posters, the average value and standard deviation for each poster is given in the last two rows. It can be observed clearly that the MI values for tested poster 7 (with six AOIs) are much larger for all participants in general than for the other posters, which may indicate that the degree of dependence or correlation between AOIs of poster 7 is much stronger. We observe also that, although values of MI for different posters might be significantly different, the differences are reduced when considering the average MI value. These facts are confirmed looking at the normalized MI (see Table A5 and Table A6).

**Figure 11:**
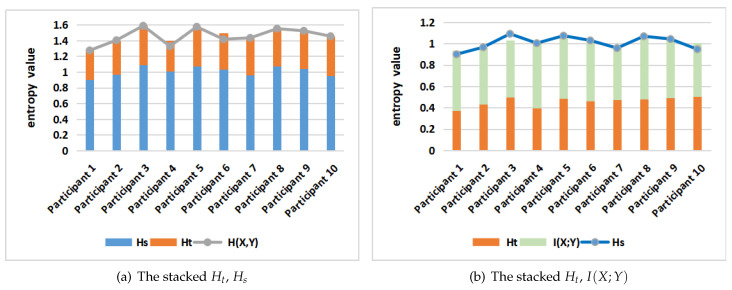
The stacked *H_t_*, *H_s_* (**a**), and *H_t_*, *I*(*X*;*Y*) (**b**), for all participants.

**Figure 12:**
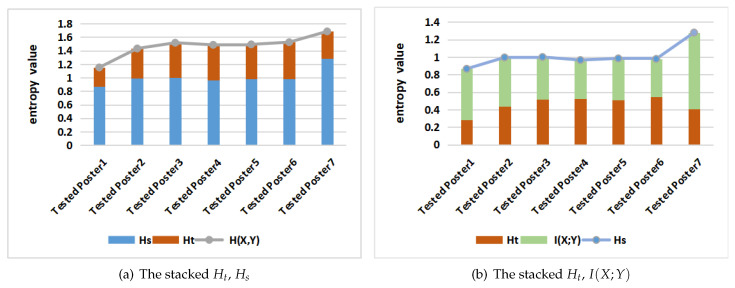
The stacked *H_t_*, *H_s_* (**a**), and *H_t_*, *I*(*X*;*Y*) (**b**), for all tested posters.

We have summarized Table A1, Table A2, Table A3 and Table A4 in Figure 11 and Figure 12. This allows readers to more intuitively observe the quantitative gaze collection of all participants. Figure 11 shows the stacked $H_{t}$, $H_{s}$, H(X,Y) and I(X;Y) in the gaze information channel from all participants when they view all tested posters. From the stacked $H_{s}$ and $H_{t}$ bar chart in Figure 11a, we see that, for every participant, the values of joint entropy H(X,Y) (marked in gray color) approximately equal the total of $H_{s}$ and $H_{t}$. Their total is equal for each separated transition matrix, Figure 11 shows that using averages is a valid approach. The joint entropy H(X,Y) measures the total uncertainty, which gives the uncertainty when every participant views the tested poster. At the same time, we can find that the values of the conditional entropy or transfer entropy $H_{t}$ (given by the crimson color bar) are close for all participants. This phenomenon illustrates, for all participants, when they begin to reading the test poster, they always like to switch between the different AOIs to better understand the context of the poster. This is consistent with the property of $H_{t}$ which reflects the randomness of gaze transition among the different AOIs.

From the right stacked $H_{t}$ and I(X;Y) chart in Figure 11, we can see that $H_{s}$ (as marked in blue color) is approximately equal to the $H_{t}$ plus I(X;Y) (see previous remark about totals). Mutual information (MI) I(X;Y) in gaze information channel represents the degree of dependence or correlation between the set of AOIs. Furthermore, $H_{s}$, which is the entropy of the equilibrium distribution π, measures how much equally the AOIs have been visited. From the blue bars in Figure 11a, it is clear that the participants 3, 5, 8, 9 spent more balanced time in each AOI when they read the tested poster since their $H_{s}$ is larger compared with the participants 1, 7, 10. This means that the participants 1, 7, 10 possibly spent more time concentrated on certain regions of the tested poster.

Figure 12 also shows the stacked $H_{t}$, $H_{s}$, H(X,Y) and I(X;Y) in gaze information channel for all tested posters. According to Figure 12, we could consider the posters into three groups, the first one with poster 1, with the lowest value of H(X,Y) and $H_{s}$, a second group with posters 2–6, with similar value of H(X,Y) and $H_{s}$, and a third one with poster 7, with highest value of H(X,Y) and $H_{s}$. Looking at Figure 1, we observe that poster 1 has one AOI that does not practically include relevant information, AOI3, this explains the lower values for this poster, as this AOI will be mostly ignored by participants. On the other extreme, poster 7 with six areas of interest is the more complex of all them. It also has the highest mutual information, and also, from Table A5 and Table A6, the highest normalized mutual information. It might mean that, although it is a more complex poster than the other ones, it is well structured and readers establish a coherent reading strategy.

Looking now at Figure 12b, we can observe the differences between the posters in the second group, from 2 to 6. All of them have similar $H_{s}$ value, but, in poster 2, the distribution is different. For poster 2, the mutual information I(X;Y) is higher (and correspondingly the entropy $H_{t}$ is lower) than for posters 3–6. This is further checked by taking a look at Table A1. It means that this poster is easier to read or to interpret than posters 3–6. It can also be seen from Table 5, where we have classed the results of the explaining the core idea stage after the experiment into two groups: expressing the core ideas basically (called basic group), and saying only some keywords (called keywords group), Table 5 gives the participants from both groups for all tested posters. Although due to the low number of participants we can not draw any conclusive result, it seems that higher mutual information in posters 2 and 7 is related to a higher cognitive comprehension. It might work in an indirect way, that is, higher MI means more coherent exploration strategies that facilitate the comprehension of the poster.

Having a look at Figure 1, we see that poster 2 contains just text in the middle AOI, being probably easier the flow from graphics to text and graphics again than in the other posters. In addition, we see that posters 4–6, although they contain four areas of interest, one of them contains very little relevant information to understand the posters, thus, although we should in principle expect more information and uncertainty with four areas than with three, the results are similar. Observe that, for the analysis of posters 2–6, we do not need to consider the normalized mutual information, as we had to do in Section 6.2.2, as we compare posters with similar values of $H_{s}$.

## 7. Discussion

We consider the information channel metrics as complementary to classic metrics for eye tracking. Actually, the information channel models the eye tracking process from an information theoretic perspective, extending the Markov chain model introduced by Krejtz et al. [44,45], and reviewed in [31]. The information channel interpretation of data communication has been successful in many areas of science, and specifically in visual computing, and we believe it also has a place in understanding eye tracking.

In particular, as already observed for Markov chain model, for stationary entropy $H_{s}$ and transition entropy $H_{t}$, greater stationary entropy $H_{s}$ means that the participant visited more equally the AOIs, while higher transition entropy $H_{t}$ denotes more uncertainty and more frequent transition between AOIs. In terms of reading a poster, it can give information on the strategy of an observer. With only $H_{s}$ and $H_{t}$, which are the metrics for the Markov channel, it is difficult to discriminate the behaviour of observers. Our model introduces the additional metrics H(X,Y), H(Y|x), I(X;Y), and I(x;Y), interpreted as the total uncertainty, the uncertainty from a given AOI, the total information shared between the AOIs, and the information shared between an AOI and all AOIs, respectively. For instance, observe from Figure 10 how we can clearly differentiate the behaviour of two observers, by using H(Y|x) and I(x;Y), and in less amount using H(X,Y), metrics that are only available once you extend the Markov chain model of eye tracking to gaze information channel.

The information channel paradigm also has the advantage of easily clustering or classification, see Table A1, Table A2, Table A3 and Table A4, and its visualization in Figure 11 and Figure 12. Given a group of observers and a poster, the transition matrices in the information channels corresponding to one class can be averaged to obtain the information channel of the class, to help understand the behaviour of that class. However, we can also obtain the average of a single observer for the different posters, by averaging the measure values obtained. The averaged results give us hints about the behaviour of observers for poster reading, and the different difficulty of reading each poster for all the observers. We believe that, in addition to help understand the cognitive process of poster reading, clues can be gathered for improving the poster design.

One weak point of the information channel model for eye tracking trajectories is that, as in the Markov chain model, the channel depends on the AOIs defined, so that changing the areas of interest the information channel measures values change. This is the same situation encountered in [53]. However, changing AOIs does not need repeating the observations, but just recomputing to which AOI belong the hit points of gaze trajectories; thus, computing the channel for different configurations of AOIs could be straightforward. The criterion of maximizing mutual information I(X;Y) gain, or minimizing its loss, for optimal subdivision or clustering [53,55], could also be used in the gaze information channel. In this paper, we have used the sections of a poster, as defined by the poster authors, as AOIs, which we thus consider semantically meaningful, although the maximization of I(X;Y) could help further in the design of the poster sections.

## 8. Conclusions and Future Work

To find quantitative links between eye movements and cognitive comprehension, we tracked 10 observers’ eye movements for reading published posters. We model eye tracking fixation sequences between content-dependent Areas of Interests (AOIs) as a Markov chain. Furthermore, we use the fact that an irreducible and aperiodic Markov chain is a special case of information or communication channel, where input and output are the same random variable, and equal to the equilibrium distribution. Thus, the gaze transition can be modeled as a discrete information channel, the *gaze information channel*. Next, some traditional eye tracking metrics, together with the gaze entropy and mutual information of the gaze information channel are calculated to quantify cognitive comprehension for every participant. As far as we know, this is the first study to use the eye tracking technology to assess cognitive comprehension when reading a scientific poster. The present work provides insights into quantitative cognitive comprehension. Although promising, there are limitations (such as a limited number of participants) to this paper that need to be addressed in continuing work. In the future, we will continue to explore the unique significance of human visual search patterns, which need to be paired with behavioral or cognitive metrics. As MI seems to be related to coherent strategies in reading a poster, we will check the difference in the gaze channel measurements for different poster design for the same content, similar to [33]. We will study the best division in AOIs, driven by the maximum transfer of information, or MI. We will also extend the information channel paradigm to the work of Ponsoda et al. [37], that is, the Markov chain of gaze displacement directions will be extended to an information channel, as we have done here with the trajectories. In addition, our current gaze channel model does not support fixation time, thus although the number of visits given by the equilibrium distribution can be a rough approximation of spent time in each AOI, for a more complete analysis we plan to integrate the fixation time into the model.

## Figures and Tables

**Table 1:** Transition matrix before normalization of three tested poster (posters 1, 2, 3, with three Areas of Interests (AOIs)) by all participants observed. The highest values of each row, in bold, correspond to transitions within the same AOI.

Tested Posters	Tested Poster 1				Tested Poster 2				Tested Poster 3			
		AOI1	AOI2	AOI3		AOI1	AOI2	AOI3		AOI1	AOI2	AOI3
	AOI1	**419**	25	0	AOI1	**337**	42	4	AOI1	**241**	54	1
*Transition matrix*	AOI2	24	**518**	19	AOI2	41	**138**	42	AOI2	45	**537**	50
	AOI3	2	19	**64**	AOI3	6	41	**506**	AOI3	10	45	**269**

**Table 2:** Transition matrix before normalization of three tested poster (posters 4, 5, 6 with four AOIs) by all participants observed. The highest values of each row, in bold, correspond to transitions within the same AOI.

Tested Posters	Tested Poster 4					Tested Poster 5					Tested Poster 6				
		AOI1	AOI2	AOI3	AOI4		AOI1	AOI2	AOI3	AOI4		AOI1	AOI2	AOI3	AOI4
	AOI1	**50**	32	2	1	AOI1	**71**	23	1	0	AOI1	**56**	32	1	0
Transitionmatrix	AOI2	31	**253**	52	0	AOI2	19	**149**	45	0	AOI2	25	**159**	49	0
	AOI3	4	50	**636**	21	AOI3	5	43	**690**	29	AOI3	8	38	**700**	38
	AOI4	0	1	21	**38**	AOI4	1	0	31	**63**	AOI4	1	4	36	**52**

**Table 3:** Transition probability of tested poster 7 (with 6 AOIs) by participants 2 and 5.

Observers	Participant 2		Participant 5	
		AOI1 AOI2 AOI3 AOI4 AOI5 AOI6		AOI1 AOI2 AOI3 AOI4 AOI5 AOI6
	AOI1	0.750 0.125 0.000 0.000 0.125 0.000	AOI1	0.000 1.000 0.000 0.000 0.000 0.000
	AOI2	0.000 0.818 0.182 0.000 0.000 0.000	AOI2	0.111 0.333 0.556 0.000 0.000 0.000
Transitionprobability	AOI3	0.000 0.125 0.813 0.063 0.000 0.000	AOI3	0.000 0.111 0.806 0.083 0.000 0.000
	AOI4	0.000 0.000 0.000 0.750 0.000 0.250	AOI4	0.000 0.000 0.133 0.800 0.067 0.000
	AOI5	0.000 0.000 0.000 0.000 0.894 0.106	AOI5	0.000 0.000 0.000 0.000 0.778 0.222
	AOI6	0.026 0.000 0.000 0.026 0.180 0.769	AOI6	0.000 0.031 0.000 0.000 0.031 0.934

**Table 4:** Equilibrium distribution, $H_{s}$, $H_{t}$, H(Y|x), H(X,Y), I(X;Y) and I(x;Y) in gaze information channel of participants 2 and 5 for poster 7.

Observers	Participant 2	Participant 5
π	(0.056, 0.076, 0.11, 0.028, 0.458, 0.271)	(0.010, 0.088, 0.353, 0.147, 0.088, 0.314)
$H_{s}$	1.412	1.487
$H_{t}$	0.504	0.529
H(Y|x)	(0.736, 0.474, 0.602, 0.562, 0.338, 0.698)	(0.000, 0.937, 0.625, 0.628, 0.529, 0.277)
H(X,Y)	1.916	2.016
I(X;Y)	0.908	0.958
I(x;Y)	(1.851, 2.030, 1.729, 2.452, 0.498, 0.613)	(2.428, 0.965, 0.643, 1.207, 1.616, 0.961)

**Table 5:** The number of participants classed in two groups according to their answer after experiment, together with mutual information (MI), MI normalized by $H_{s}$, and MI normalized by H(X,Y).

Tested Poster	Tested Poster 1	Tested Poster 2	Tested Poster 3	Tested Poster 4	Tested Poster 5	Tested Poster 6	Tested Poster7
**Basic Group**	4	7	6	6	4	4	5
**Keywords Group**	6	3	4	4	6	6	5
MI	0.580	0.559	0.483	0.446	0.474	0.428	0.873
MI normalized by $H_{s}$	0.668	0.569	0.484	0.464	0.480	0.443	0.679
MI normalized by H(X,Y)	0.507	0.410	0.324	0.303	0.318	0.289	0.523

## Appendix A

**Table A1:** Mutual information, I(X;Y), values in gaze information channel for all posters and all participants.

MI	Tested Poster1	Tested Poster2	Tested Poster3	Tested Poster4	Tested Poster5	Tested Poster6	Tested Poster7	Average Value	Standard Deviation
Participant 1	0.422	0.489	0.584	0.474	0.548	0.474	0.716	0.530	0.091
Participant 2	0.469	0.608	0.377	0.545	0.411	0.398	0.908	0.531	0.172
Participant 3	0.782	0.557	0.533	0.394	0.512	0.428	0.969	0.596	0.191
Participant 4	0.664	0.641	0.582	0.411	0.351	0.587	1.043	0.611	0.207
Participant 5	0.655	0.542	0.422	0.415	0.671	0.449	0.958	0.587	0.180
Participant 6	0.497	0.786	0.301	0.429	0.544	0.481	0.956	0.571	0.208
Participant 7	0.575	0.508	0.512	0.439	0.464	0.294	0.609	0.486	0.095
Participant 8	0.638	0.544	0.611	0.412	0.452	0.406	1.062	0.589	0.211
Participant 9	0.512	0.498	0.470	0.427	0.497	0.397	1.023	0.546	0.198
Participant 10	0.581	0.415	0.437	0.514	0.288	0.370	0.484	0.441	0.089
Average Value	0.580	0.559	0.483	0.446	0.474	0.428	0.873	–	–
Standard Deviation	0.103	0.097	0.095	0.047	0.103	0.074	0.189	–	–

**Table A2:** $H_{s}$ values in gaze information channel for all posters and all participants.

$H_{s}$	Tested Poster1	Tested Poster2	Tested Poster3	Tested Poster4	Tested Poster5	Tested Poster6	Tested Poster7	Average Value	Standard Deviation
Participant 1	0.711	0.833	0.939	0.930	1.021	0.924	0.948	0.901	0.093
Participant 2	0.889	0.942	0.744	1.15	0.755	0.88	1.412	0.967	0.221
Participant 3	1.008	1.051	1.077	1.087	1.022	1.115	1.293	1.093	0.088
Participant 4	0.904	0.936	1.061	0.903	0.930	0.980	1.323	1.005	0.139
Participant 5	0.851	1.025	1.050	0.970	1.229	0.915	1.487	1.075	0.201
Participant 6	0.919	0.918	0.941	0.930	1.023	1.128	1.360	1.031	0.152
Participant 7	0.836	0.998	1.030	0.918	0.929	0.999	1.004	0.959	0.063
Participant 8	0.914	1.066	1.011	0.808	1.184	1.120	1.386	1.070	0.174
Participant 9	0.798	1.092	1.055	0.996	1.015	0.780	1.569	1.044	0.242
Participant 10	0.822	1.093	1.094	0.959	0.727	0.934	1.009	0.948	0.126
Average Value	0.865	0.995	1.000	0.965	0.984	0.978	1.279	–	–
Standard Deviation	0.077	0.082	0.099	0.091	0.152	0.109	0.206	–	–

**Table A3:** $H_{t}$ values in gaze information channel for all posters and all participants.

$H_{t}$	Tested Poster1	Tested Poster2	Tested Poster3	Tested Poster4	Tested Poster5	Tested Poster6	Tested Poster7	Average Value	Standard Deviation
Participant 1	0.289	0.344	0.355	0.456	0.473	0.467	0.232	0.374	0.088
Participant 2	0.420	0.333	0.367	0.616	0.344	0.466	0.504	0.436	0.094
Participant 3	0.226	0.494	0.543	0.693	0.509	0.687	0.324	0.497	0.160
Participant 4	0.240	0.295	0.478	0.492	0.580	0.393	0.280	0.394	0.118
Participant 5	0.196	0.483	0.627	0.555	0.558	0.453	0.529	0.486	0.129
Participant 6	0.422	0.132	0.639	0.505	0.479	0.647	0.404	0.461	0.161
Participant 7	0.261	0.491	0.518	0.480	0.465	0.704	0.396	0.474	0.123
Participant 8	0.276	0.523	0.398	0.396	0.732	0.714	0.324	0.480	0.169
Participant 9	0.286	0.592	0.582	0.569	0.518	0.383	0.537	0.495	0.108
Participant 10	0.238	0.678	0.658	0.445	0.439	0.557	0.525	0.506	0.139
Average Value	0.285	0.437	0.517	0.521	0.510	0.547	0.406	–	–
Standard Deviation	0.073	0.152	0.108	0.084	0.097	0.124	0.108	–	–

**Table A4:** H(X,Y) values in gaze information channel of all posters and all participants.

*H(X,Y)*	Tested Poster1	Tested Poster2	Tested Poster3	Tested Poster4	Tested Poster5	Tested Poster6	Tested Poster7	Average Value	Standard Deviation
Participant 1	1.000	1.176	1.293	1.385	1.494	1.391	1.180	1.274	0.155
Participant 2	1.310	1.275	1.111	1.765	1.099	1.346	1.916	1.403	0.293
Participant 3	1.234	1.544	1.620	1.781	1.532	1.802	1.617	1.590	0.175
Participant 4	1.144	1.231	1.539	1.395	1.510	1.373	1.603	1.399	0.155
Participant 5	1.047	1.508	1.678	1.526	1.787	1.367	2.016	1.561	0.287
Participant 6	1.341	1.050	1.580	1.435	1.502	1.775	1.764	1.492	0.234
Participant 7	1.097	1.489	1.548	1.398	1.393	1.703	1.400	1.433	0.172
Participant 8	1.190	1.589	1.409	1.204	1.915	1.835	1.710	1.550	0.270
Participant 9	1.083	1.683	1.637	1.566	1.532	1.164	2.015	1.539	0.316
Participant 10	1.060	1.771	1.752	1.404	1.166	1.492	1.534	1.454	0.250
Average Value	1.151	1.432	1.517	1.486	1.493	1.525	1.685	–	–
Standard Deviation	0.109	0.224	0.184	0.170	0.232	0.223	0.268	–	–

**Table A5:** Normalized mutual information by $H_{s}$ in gaze information channel of all posters and all participants.

Normalized MI	Tested Poster1	Tested Poster2	Tested Poster3	Tested Poster4	Tested Poster5	Tested Poster6	Tested Poster7	Average Value	Standard Deviation
Participant 1	0.594	0.587	0.622	0.510	0.537	0.513	0.755	0.588	0.079
Participant 2	0.528	0.645	0.507	0.474	0.544	0.452	0.643	0.542	0.071
Participant 3	0.776	0.530	0.495	0.362	0.501	0.384	0.749	0.542	0.151
Participant 4	0.735	0.685	0.549	0.455	0.377	0.599	0.788	0.598	0.138
Participant 5	0.770	0.529	0.402	0.428	0.546	0.491	0.644	0.544	0.118
Participant 6	0.541	0.856	0.320	0.461	0.532	0.426	0.703	0.548	0.166
Participant 7	0.688	0.509	0.497	0.478	0.499	0.294	0.607	0.510	0.113
Participant 8	0.698	0.510	0.604	0.510	0.382	0.363	0.766	0.548	0.141
Participant 9	0.642	0.456	0.445	0.429	0.490	0.509	0.652	0.517	0.086
Participant 10	0.707	0.380	0.399	0.536	0.396	0.396	0.480	0.471	0.110
Average Value	0.668	0.569	0.484	0.464	0.480	0.443	0.679	–	–
Standard Deviation	0.084	0.127	0.090	0.048	0.065	0.084	0.089	–	–

**Table A6:** Normalized mutual information by H(X,Y) in gaze information channel of all posters and all participants.

Normalized MI	Tested Poster1	Tested Poster2	Tested Poster3	Tested Poster4	Tested Poster5	Tested Poster6	Tested Poster7	Average Value	Standard Deviation
Participant 1	0.422	0.416	0.452	0.342	0.367	0.341	0.607	0.421	0.092
Participant 2	0.358	0.477	0.339	0.309	0.374	0.296	0.474	0.375	0.073
Participant 3	0.634	0.361	0.329	0.221	0.334	0.238	0.599	0.388	0.165
Participant 4	0.580	0.521	0.378	0.295	0.232	0.428	0.651	0.441	0.152
Participant 5	0.626	0.359	0.251	0.272	0.375	0.328	0.475	0.384	0.130
Participant 6	0.371	0.749	0.191	0.299	0.362	0.271	0.542	0.398	0.189
Participant 7	0.524	0.341	0.331	0.314	0.333	0.173	0.435	0.350	0.109
Participant 8	0.536	0.342	0.434	0.342	0.236	0.221	0.621	0.390	0.149
Participant 9	0.473	0.296	0.287	0.273	0.324	0.341	0.508	0.357	0.094
Participant 10	0.548	0.234	0.249	0.366	0.247	0.248	0.316	0.315	0.113
Average Value	0.507	0.410	0.324	0.303	0.318	0.289	0.523	–	–
Standard Deviation	0.099	0.145	0.083	0.042	0.058	0.074	0.103	–	–
